# Professional Identity, Psychological Resilience, and Adaptive Performance in Junior Nurses Exposed to Workplace Bullying: A Latent Profile and Network Analysis

**DOI:** 10.1155/jonm/6681049

**Published:** 2026-10-29

**Authors:** Lanjun Luo, Huilan Shu, Xueyan Wang, Fengling Li, Yuze Wu, Siyuan Li, Min Tan, Xuemei Wei

**Affiliations:** ^1^ Key Laboratory of Digital-Intelligent Disease Surveillance and Health Governance School of Management North Sichuan Medical College Nanchong 637100 China nsmc.edu.cn; ^2^ Affiliated Hospital of North Sichuan Medical College Nanchong 637000 China hospital-nsmc.com.cn; ^3^ Department of General Surgery Sichuan Mianyang 404 Hospital Mianyang 621000 China

**Keywords:** adaptive performance, junior nurses, latent profile analysis, network analysis, professional identity, psychological resilience, workplace bullying

## Abstract

**Aim:**

To identify resource‐adaptation profiles and profile‐specific network organization among junior nurses exposed to workplace bullying by examining professional identity (PID), psychological resilience (PSR), and adaptive performance (ADP).

**Design:**

A multicenter, cross‐sectional study.

**Methods:**

Survey data were collected from 7231 junior nurses in 90 tertiary hospitals in Sichuan Province, China; 5839 met the workplace bullying exposure criterion and were included. Latent profile analysis was performed using 36 item‐level indicators of PID, PSR, and ADP. Ordered logistic regression examined factors associated with profile membership, and network analysis compared profile‐specific resource organization. Robustness was assessed using network comparison tests, residualized networks, hospital‐cluster robust sensitivity analysis, and bootstrap stability analyses.

**Results:**

Three profiles were identified: the resource‐constrained adaptive profile (*n* = 1906, 32.64%), moderate resource‐adaptation profile (*n* = 3152, 53.98%), and high resource‐adaptation profile (*n* = 781, 13.38%). More frequent night shifts, longer weekly working hours, poorer sleep quality, and lack of regular exercise were associated with greater odds of membership in a more resource‐constrained profile. The high resource‐adaptation profile showed the highest global network strength (16.223) and the largest mean absolute nonzero edge weight (0.086), despite fewer nonzero edges. PID2 (Social confidence), PID8 (Social interaction), PSR2 (Achievement pride), and PSR3 (Calm handling) repeatedly served as bridge nodes. Bootstrap analyses supported centrality stability, with CS coefficients of 0.750 across base and residualized networks.

**Conclusion:**

Junior nurses exposed to workplace bullying showed heterogeneous resource configurations and profile‐specific network organization. Integrating latent profile and network analyses showed how PID, PSR, and ADP may be configured and interconnected under a resource‐threatening work context.

## 1. Introduction

Workplace bullying has emerged as an important occupational health and workforce problem in healthcare settings [1, 2]. It is commonly understood as repeated exposure to negative acts, including psychological, verbal, or work‐related mistreatment, in situations where the target may have difficulty defending themselves [3]. Unlike violence initiated by patients or family members, workplace bullying is embedded in workplace relationships and organizational routines, making it difficult to recognize, report, and manage [4]. Previous studies have shown that such mistreatment can weaken nurses’ perceived safety, belonging, and professional identity (PID), while increasing anxiety, depression, burnout, and turnover intention among nursing staff [5, 6]. These consequences may threaten workforce stability and patient care quality, particularly in clinical environments where teamwork and continuity of care are essential.

Junior nurses may be especially vulnerable because they have limited clinical experience, fewer coping resources, and professional identities that are still developing [7]. This vulnerability is shaped by the early stage of professional socialization. Junior nurses are still consolidating their clinical confidence, role expectations, team relationships, and sense of organizational belonging. Compared with experienced nurses, they may have fewer accumulated coping strategies, less familiarity with informal workplace norms, and lower bargaining power in hierarchical clinical environments. Exposure to workplace bullying during this period may, therefore, be associated with psychological distress, weakened PID formation, and poorer occupational adaptation [8]. Understanding how junior nurses maintain and organize internal resources after such exposure is relevant to early‐career workforce development and retention.

Research on workplace violence and bullying in nursing has mainly examined adverse psychological and behavioral outcomes, including anxiety, depression, post‐traumatic stress symptoms, burnout, job stress, and turnover intention [9]. Network analysis has also been used to examine how negative symptoms and work‐related outcomes are connected after workplace adversity. For example, previous studies have investigated associations among workplace violence, burnout, depressive symptoms, suicidality, and turnover intention in healthcare workers, as well as symptom networks of anxiety, depression, and post‐traumatic stress among nurses exposed to workplace mistreatment [10, 11]. These studies have improved understanding of maladaptive responses. However, less is known about the positive internal resources that may support continued professional functioning after workplace bullying. Adaptation in this context cannot be fully explained by distress‐related variables alone because nurses may differ in how professional, psychological, and behavioral resources are preserved and organized.

Conservation of Resources (COR) theory provides a suitable framework for examining this issue [12, 13]. The theory proposes that stress arises when valued resources are threatened, lost, or insufficiently replenished. Workplace bullying can, therefore, be viewed as a resource‐threatening work condition in which interpersonal mistreatment challenges resources related to professional belonging, self‐regulation, and adaptive functioning. Under such conditions, junior nurses’ adaptation may depend on the level of available resources and whether these resources remain fragmented, moderately maintained, or more strongly integrated. This perspective directs attention from isolated psychological variables to broader patterns of resource configuration.

Within this resource‐oriented framework, PID, psychological resilience (PSR), and adaptive performance (ADP) represent interrelated domains of occupational adaptation. PID provides an identity anchor through which junior nurses derive professional value, role belonging, social recognition, and commitment to nursing [14]. In the context of workplace bullying, this resource is especially relevant because exclusion, humiliation, unfair criticism, or devaluation may challenge nurses’ sense of professional belonging [15]. PSR reflects the capacity to maintain emotional regulation, perceived control, and problem solving under adversity, thereby supporting the preservation of internal resources under stressful interpersonal conditions [16]. ADP represents the behavioral expression of adaptation, including maintaining composure under pressure, responding flexibly to urgent demands, and adjusting to interpersonal and organizational complexity [17]. Together, these constructs form a resource‐adaptation system that links professional self‐definition, self‐regulatory capacity, and adaptive work behavior.

A resource‐adaptation perspective requires an analytic approach that can capture both heterogeneity between nurses and the organization of resources within nurses. Traditional variable‐centered analyses can estimate average associations among PID, PSR, and ADP, but they provide limited information about whether these resources co‐occur in distinct patterns across individuals. For junior nurses exposed to workplace bullying, an important question is whether internal resources form qualitatively different profiles, such as constrained, moderate, or more favorable resource‐adaptation patterns. Latent profile analysis (LPA) addresses this question by identifying subgroups with distinct configurations across multiple resource indicators, thereby complementing conventional total‐score approaches [18, 19].

Profile membership alone, however, does not indicate how resources are connected within each subgroup. Two profiles may differ in mean resource levels, but they may also differ in the strength, selectivity, and cross‐domain organization of associations among specific indicators. Network analysis can, therefore, extend person‐centered findings by estimating the conditional association structure of PID, PSR, and ADP within each latent profile [20, 21]. From a COR perspective, higher global strength may indicate closer resource integration, bridge expected influence may identify indicators linking identity‐related, resilience‐related, and ADP domains, and negative edges may provide preliminary evidence of possible resource tensions or trade‐offs [22].

To address these issues, this study integrated LPA and network analysis to examine resource‐adaptation patterns among junior nurses exposed to workplace bullying. The aims were to (1) identify latent profiles based on item‐level indicators of PID, PSR, and ADP; (2) examine demographic, work‐related, and health‐related factors associated with profile membership; (3) compare profile‐specific network structures, including global strength, node centrality, bridge expected influence, and network invariance; and (4) evaluate the robustness of the findings through regression diagnostics, hospital‐cluster sensitivity analysis, residualized networks, and bootstrap stability analyses.

## 2. Methods

### 2.1. Study Design, Participants, and Ethical Considerations

This multicenter cross‐sectional study was guided by the STROBE reporting standards. Data were collected between September 2021 and October 2022 from junior registered nurses in 90 tertiary‐level hospitals in Sichuan Province, China. Junior nurses were operationally defined as registered nurses with five or fewer years of professional experience and no prior formal employment history before entering their current hospital, excluding clinical internships.

The inclusion criteria were (1) total working duration of 5 years or less; (2) possession of a valid nursing license; (3) voluntary participation with electronic informed consent; (4) age 18 years or older; and (5) exposure to workplace bullying, as determined by the criterion described in Section 2.2.2. Exclusion criteria included major stressful events during the preceding 6 months, such as death of an immediate family member, major surgery, or serious illness, and current use of psychotropic medication or psychotherapy.

A total of 7231 junior registered nurses were recruited. Among them, 5839 nurses met the workplace bullying exposure criterion and were included in the final analytic sample. Participants were required to provide a valid response to each questionnaire item before submission; therefore, no item‐level missing data were retained in the analytic dataset.

This study was approved by the Ethics Committee of the Affiliated Hospital of North Sichuan Medical College (Approval No. 2021ER006‐1). All participants signed electronic informed consent voluntarily before participation. Participant privacy was protected throughout the study. Personal data were collected solely for research purposes and stored securely on password‐protected servers with access restricted to the research team. All personally identifiable information was destroyed upon the study’s completion to ensure long‐term confidentiality. Each research team member signed a formal confidentiality agreement to guarantee ethical handling of sensitive information.

### 2.2. Measures

#### 2.2.1. The General Information Questionnaire

The general information questionnaire for junior nurses included 21 variables: age, gender, household registration, marital status, number of cohabiting family members, religious affiliation, educational background, hospital level, monthly individual income, monthly per capita family income, years of work experience, weekly day shift frequency, weekly night shift frequency, average daily sleep duration, subjective sleep quality, history of occupational exposure (e.g., needle stick injury or radiation), smoking status, alcohol consumption, regular physical exercise, chronic disease diagnosis in the past year, and history of childhood parental absence due to labor migration.

#### 2.2.2. The Six‐Item Workplace Bullying Scale

Workplace bullying was assessed using six items adapted from Attell et al. [23]. The items were translated and culturally adapted for the present Chinese nursing sample. The six items assess distinct negative workplace behaviors: excessive work demands, close supervision, teasing or insults, extra workload, being undervalued by supervisors, and unfair humiliation. Items are rated on a 5‐point Likert scale (1 = *rarely occurs* and 5 = *frequently occurs*), yielding total scores from 6 to 30. Higher scores reflect greater exposure to workplace bullying. In this study, exposure was defined as a score of ≥ 3 (“sometimes” or more often) on at least one item. This item‐level criterion was used to capture behaviorally diverse bullying experiences and to avoid excluding nurses who face concentrated but meaningful exposure to a specific negative act. It also aligns with prior workplace bullying research that uses exposure to at least one negative act as a classification criterion when no universally accepted cutoff exists [24]. The original study reported a Cronbach’s *α* of 0.81, and the Cronbach’s *α* in the present study was 0.812. The scale also showed acceptable construct validity in the present sample: KMO = 0.810, Bartlett’s test *χ* ^2^ = 3391.273, and *p* < 0.001; exploratory factor analysis extracted one factor explaining 53.958% of the variance; and CFA showed *χ* ^2^ = 139.488, df = 6, RMSEA = 0.087, CFI = 0.995, TLI = 0.987, and SRMR = 0.023.

#### 2.2.3. The Nine‐Item Professional Identity Scale

The Professional Identity Scale [25] was used to evaluate nurses’ PID. It contains nine items rated on a 5‐point Likert scale, with total scores ranging from 9 to 45. Higher scores show stronger PID. The original study reported a Cronbach’s *α* of 0.938, and the Cronbach’s *α* in the present study was 0.908. The scale also showed acceptable construct validity in the present sample: KMO = 0.912, Bartlett’s test *χ* ^2^ = 7489.275, and *p* < 0.001; exploratory factor analysis extracted two factors explaining 69.357% of the variance; and CFA showed CFI = 0.982, TLI = 0.972, and SRMR = 0.031.

#### 2.2.4. The Fourteen‐Item Psychological Resilience Scale

PSR was measured with the Chinese version of the 14‐item Resilience Scale [26]. Each item was rated on a 5‐point Likert scale, and the total score ranged from 14 to 70. Higher scores indicate better PSR. Previous studies reported a Cronbach’s *α* of 0.928, and the Cronbach’s *α* in the present study was 0.935. The scale showed acceptable construct validity in the present sample: KMO = 0.951, Bartlett’s test *χ* ^2^ = 12,415.856, and *p* < 0.001; exploratory factor analysis extracted two factors explaining 61.883% of the variance; and CFA showed CFI = 0.976, TLI = 0.971, and SRMR = 0.037.

#### 2.2.5. The Thirteen‐Item Adaptive Performance Scale

ADP was measured using selected dimensions from the scale developed by Tao and Wang [27]. The original scale includes four dimensions: stress and emergency response, innovative problem solving, continuous learning at work, and cultural‐interpersonal facilitation. In the present study, two dimensions were used: stress and emergency response and cultural‐interpersonal facilitation. These two dimensions were selected because the study focused on internal resource‐adaptation configurations among junior nurses exposed to workplace bullying. Stress and emergency response reflect nurses’ ability to regulate emotions and respond effectively to high‐pressure, urgent, or unexpected clinical demands, whereas cultural‐interpersonal facilitation reflects their ability to adapt to interpersonal and team‐based work contexts. These two dimensions are, therefore, closely aligned with the occupational adaptation processes most relevant to workplace bullying exposure, which is embedded in workplace interactions and may be associated with nurses’ emotional, interpersonal, and clinical coping resources. The scale included 13 items rated on a 5‐point Likert scale, with total scores between 13 and 65. Higher scores indicate stronger ADP. The Cronbach’s *α* in the present study was 0.944. The scale showed acceptable construct validity in the present sample: KMO = 0.926, Bartlett’s test *χ* ^2^ = 13,534.472, and *p* < 0.001; exploratory factor analysis extracted two factors explaining 67.900% of the variance; and CFA showed CFI = 0.974, TLI = 0.967, and SRMR = 0.048.

Because all study variables were measured using self‐report questionnaires, common method bias was assessed using Harman’s single‐factor test. Six factors with eigenvalues greater than 1 were extracted without rotation, and the first factor explained 38.005% of the total variance, suggesting that common method bias was not a severe concern in this study. More details about the questionnaires used in this study and the brief meanings of variables can be found in Table S1.

### 2.3. Sample Size

Sample size adequacy was evaluated for both LPA and profile‐specific network analysis. Because there is no single universally accepted power formula for exploratory LPA with item‐level indicators, adequacy was assessed using several practical criteria: each retained profile should include at least 50 participants; the smallest profile should account for at least 5% of the analytic sample to support substantive interpretation; and the total sample size should be sufficient relative to the number of observed indicators [28].

To examine the network structure of three key resource‐adaptation domains, namely, PSR, PID, and ADP, among junior nurses exposed to workplace bullying, a total of 36 variables were included in the network model (9 for PID, 14 for PSR, and 13 for ADP). The initial cleaned dataset contained 7231 junior nurses with no missing values in the variables used for this analysis. Of these, 5839 met the workplace bullying exposure criterion and formed the final analytic sample. In the retained three‐profile solution, the smallest profile included 781 participants, representing 13.4% of the analytic sample. Thus, the sample size met the predefined adequacy criteria for LPA and supported the interpretability of all retained profiles.

Network sample size adequacy was further evaluated at the profile level because networks were estimated separately within each latent profile. Following recommendations for psychological network estimation, the number of parameters for a network with *k* nodes can be approximated as *k* + *k* × (*k* − 1)/2, including node parameters and unique pairwise associations [21]. With 36 nodes, this yielded 666 parameters. Even the smallest latent profile contained 781 participants, exceeding this parameter‐count benchmark for profile‐specific network estimation.

### 2.4. Statistical Analysis

#### 2.4.1. LPA

LPA was conducted to examine heterogeneity in the resource‐adaptation profiles of junior nurses who had experienced workplace bullying. The model was estimated using Mplus (Version 8.3), with 36 continuous observed indicators. Item‐level indicators were used rather than total scale scores to preserve within‐construct heterogeneity and to ensure consistency with the nodes included in the subsequent network analysis [29, 30]. This approach also avoided the additional assumptions imposed by sum scoring, which may obscure item‐specific variation [31]. Because all indicators used 5‐point Likert‐type response formats, the items were entered on their original metric and treated as approximately continuous. This treatment is consistent with original quantitative applications of workplace bullying measures [23] and with methodological evidence suggesting that ordered variables with five or more categories can often be treated as approximately continuous under appropriate conditions [32]. The implications of this analytic choice were addressed in the limitations section.

Model estimation began with a one‐profile solution and proceeded sequentially to a six‐profile solution. To reduce the risk of local maxima, 200 random starts and 20 optimization iterations were specified. Model fit was evaluated using Akaike Information Criterion (AIC), Bayesian Information Criterion (BIC), adjusted BIC (aBIC), entropy, the Lo–Mendell–Rubin (LMR) adjusted likelihood ratio test, and the Bootstrap Likelihood Ratio Test (BLRT). Lower information criteria indicated better fit, entropy closer to one indicated better classification accuracy, and significant LMR and BLRT values (*p < *0.05) suggested improvement over the model with one fewer profile [18]. Average posterior probabilities were also inspected to assess classification certainty. The final profile solution was selected based on statistical fit, classification quality, profile size, substantive interpretability, and consistency with the resource‐adaptation framework.

#### 2.4.2. Demographic Analysis

Baseline characteristics of the three latent profile groups were analyzed using the *tableone* (Version 0.9.5) Python package [33]. Continuous variables were reported as mean ± standard deviation when normally distributed or as medians with interquartile ranges when nonnormally distributed. Categorical variables were summarized using frequencies and percentages. Normality was assessed using the *scipy.stats.normaltest* function in the *SciPy* (Version 1.11.3) Python package [34], with a significance threshold set at *α* = 0.05. Differences between latent groups were examined using the Kruskal–Wallis test for continuous variables and the chi‐square test for categorical variables.

To identify key factors associated with profile membership, ordinal logistic regression was performed using the *OrderedModel* method from the *Statsmodels* (Version 0.14.4) Python package [35]. Continuous predictors were standardized using *z-scores* before entering the regression model, and categorical predictors were dummy‐coded. The proportional‐odds assumption was evaluated using a global Brant‐style parallelism test. For ordered logistic regression, latent profile membership was reverse‐coded so that higher outcome values represented membership in a more resource‐constrained profile; therefore, odds ratios (ORs) greater than one indicated higher odds of belonging to a more resource‐constrained profile. Results were presented as ORs with 95% confidence intervals. All predictors from the ordered logistic model were displayed in the forest plot, and variables with *p* < 0.05 were selected as covariates for the residualized network sensitivity analyses.

To examine whether the ordered specification influenced the findings, multinomial logistic regression was conducted as a sensitivity analysis. Because nurses were recruited from multiple hospitals, an additional sensitivity analysis using hospital‐cluster robust standard errors was performed. This analysis was restricted to participants with valid hospital‐cluster information, resulting in a reduced analytic sample of 5682 nurses from 89 hospitals.

#### 2.4.3. Network Analysis

This study applied network analysis using R (Version 4.4.1) to explore the interrelations among PID, PSR, and ADP in junior nurses exposed to workplace bullying. The network comprised 36 nodes, including nine indicators of PID, 14 of PSR, and 13 of ADP. Consistent with the LPA specification, 5‐point Likert‐type items were treated as approximately continuous variables in the primary network models. Network estimation and visualization were conducted separately for each latent profile using the *estimateNetwork* function and *qgraph* (Version 1.9.8) packages. The Extended Bayesian Information Criterion Graphical Lasso (EBICglasso) method was employed to estimate the networks [21]. This approach combines the Extended BIC with Lasso regularization to detect partial correlations among variables, allowing for the construction of sparse and stable network structures. Nodes in the resulting graphs represent specific psychological dimensions, and edges denote associations between them. Positive associations were indicated by blue solid lines, whereas negative associations were represented by red dashed lines. The thickness of the edges reflected the strength of the relationships.

To identify influential nodes, two centrality indices were calculated: strength centrality and expected influence. Strength centrality measured the total absolute weight of connections for each node, while expected influence accounted for both the direction and magnitude of these connections. Additionally, bridge centrality indices were calculated using the *networktools* (Version 1.6.0) package to determine which nodes served as bridges between different psychological domains. These included bridge strength and bridge expected influence.

Network stability was evaluated using two bootstrap procedures implemented in the *bootnet* package (Version 1.6). First, case‐dropping bootstrap analysis with 1000 iterations was used to assess the stability of centrality indices. The case‐dropping stability coefficient (CS coefficient) was calculated to quantify the maximum proportion of cases that could be dropped while retaining a correlation of at least 0.70 between the original and subset‐based centrality estimates. Second, nonparametric bootstrap analysis with 1000 iterations was used to estimate 95% confidence intervals for edge weights and to evaluate the precision of the estimated network structure.

Network structures were then compared across latent profiles using a permutation‐based framework implemented via the *NetworkComparisonTest* (Version 2.2.2) package. This approach assessed differences in overall network structure and global strength. Each pairwise comparison used 1000 permutations. For edge‐level comparisons, the Benjamini–Hochberg procedure was applied to control the false‐discovery rate [36].

In addition to the primary networks estimated from raw item scores, residualized network models were estimated as a sensitivity analysis. Each of the 36 psychological indicators was separately regressed on covariates significantly associated with profile membership in the ordered logistic regression, and the resulting residuals were used as adjusted network nodes. This approach was adopted to examine whether the primary network patterns remained stable after accounting for demographic and work‐related covariates.

## 3. Results

### 3.1. LPA

Based on the LPA results shown in Table 1, the three‐class solution was identified as the optimal model. Although models with four to six classes showed further reductions in AIC, BIC, and aBIC, these more complex solutions produced smaller and more fragmented subgroups without substantially changing the broad low‐to‐high resource‐adaptation gradient. Therefore, considering model parsimony, classification quality, subgroup size, and substantive interpretability, the three‐profile solution was retained. This model yielded an AIC of 392,451.468, a BIC of 393,425.626, and an aBIC of 392,961.681. It also demonstrated high classification accuracy, with an entropy value of 0.952. Results from the LMR adjusted likelihood ratio test and the BLRT were statistically significant, further supporting the three‐class model over the two‐class alternative. As shown in Table S2, the diagonal average posterior probabilities were 0.975, 0.981, and 0.971 for Classes 1–3, respectively, indicating good classification certainty.

**TABLE 1:** Comparison of latent profile models’ goodness of fit.

Classes	AIC	BIC	aBIC	Entropy	LMR (*p*)	BLRT (*p*)	Proportion (%)
1	471,466.997	471,947.404	471,718.609	—	—	—	—
2	413,448.043	414,175.325	413,828.954	0.945	< 0.001	< 0.001	49.426/50.574
3	392,451.468	393,425.626	392,961.681	0.952	< 0.001	< 0.001	32.643/53.982/13.375
4	380,360.281	381,581.315	380,999.794	0.944	< 0.001	< 0.001	16.578/35.280/9.214/38.928
5	375,632.994	377,100.904	376,401.807	0.949	< 0.001	< 0.001	6.217/11.663/34.201/38.671/9.248
6	371,463.263	373,178.048	372,361.376	0.955	< 0.001	< 0.001	5.926/11.440/33.413/6.816/34.783/7.622

Figure 1 illustrates the average item‐level scores across subdimensions for the three identified latent profiles. As further summarized in Table 2, the profiles showed a monotonic gradient across PID, PSR, and ADP. Class 1 comprised 1906 nurses (32.64%) and demonstrated consistently lower mean scores in PID, PSR, and ADP than the other two profiles. The mean item scores for this profile were 3.051 for PID, 2.922 for PSR, and 3.314 for ADP, indicating a comparatively constrained internal resource‐adaptation pattern. This class was labeled the “resource‐constrained adaptive” profile. Class 2, which accounted for 3152 participants (53.98%), represented a “moderate resource‐adaptation” profile. Members of this class exhibited intermediate mean scores across all three psychological domains, with mean item scores of 3.742 for PID, 3.620 for PSR, and 3.894 for ADP. As the largest subgroup, it reflected a moderate and relatively balanced resource‐adaptation pattern. Class 3 consisted of nurses with the most favorable profile, labeled “high resource‐adaptation” profile. This group included 781 participants (13.38%) and showed the highest mean scores in PID, PSR, and ADP, with corresponding mean item scores of 4.457, 4.375, and 4.527, respectively.

**FIGURE 1:**
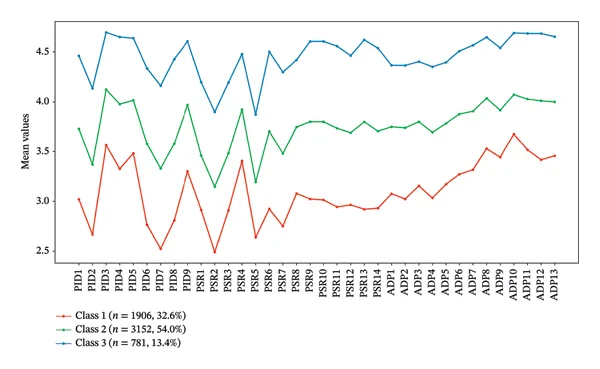
Three‐class LPA profile plot.

**TABLE 2:** Mean (SD) PID, PSR, and ADP scores across the three latent profiles.

Class	Label	*N*	%	PID, mean (SD)	PSR, mean (SD)	ADP, mean (SD)
1	Resource‐constrained adaptive profile	1906	32.643	3.051 (0.468)	2.922 (0.350)	3.314 (0.382)
2	Moderate resource‐adaptation profile	3152	53.982	3.742 (0.380)	3.620 (0.320)	3.894 (0.230)
3	High resource‐adaptation profile	781	13.375	4.457 (0.460)	4.375 (0.370)	4.527 (0.372)

### 3.2. Factors Associated With Latent Profile Membership

Univariate analyses were conducted to compare characteristics across the three latent profile groups, as shown in Table 3. Significant differences were observed in age (*H* = 6.275, *p* = 0.0434), family income (*H* = 11.300, *p* = 0.0035), individual income (*H* = 6.565, *p* = 0.0375), frequency of night shifts (*H* = 28.153, *p* < 0.001), sleep duration (*H* = 98.866, *p* < 0.001), gender (*χ* ^2^ = 9.886, *p* = 0.0071), childhood parental absence due to labor migration (*χ* ^2^ = 29.736, *p* < 0.001), marital status (*χ* ^2^ = 13.362, *p* = 0.0096), educational background (*χ* ^2^ = 32.913, *p* < 0.001), occupational exposure (*χ* ^2^ = 171.665, *p* < 0.001), weekly working hours (*χ* ^2^ = 72.886, *p* < 0.001), sleep quality (*χ* ^2^ = 352.862, *p* < 0.001), exercise habits (*χ* ^2^ = 299.098, *p* < 0.001), and chronic disease status (*χ* ^2^ = 21.802, *p* < 0.001). No significant between‐profile differences were observed for the number of cohabiting family members, day‐shift frequency, household registration, religious affiliation, hospital level, alcohol consumption, or smoking status.

**TABLE 3:** Comparison of participant characteristics across latent groups.

Variable	Class 1	Class 2	Class 3	Statistic	*p*
Age	23.00 (22.00, 24.00)	23.00 (22.00, 24.00)	23.00 (22.00, 24.00)	*H* = 6.275	0.0434
Family member	4.00 (3.00, 5.00)	4.00 (3.00, 5.00)	4.00 (3.00, 5.00)	*H* = 3.345	0.1878
Family income	3000.00 (2000.00, 4000.00)	3000.00 (2000.00, 4000.00)	3000.00 (2000.00, 5000.00)	*H* = 11.300	0.0035
Income	2200.00 (2000.00, 3000.00)	2300.00 (2000.00, 3000.00)	2300.00 (2000.00, 3000.00)	*H* = 6.565	0.0375
Day shift	3.00 (3.00, 5.00)	3.00 (3.00, 5.00)	3.00 (3.00, 4.00)	*H* = 1.378	0.502
Night shift	2.00 (1.00, 2.00)	2.00 (1.00, 2.00)	2.00 (1.00, 2.00)	*H* = 28.153	< 0.001
Sleep time	7.00 (6.00, 7.00)	7.00 (6.00, 7.50)	7.00 (6.50, 8.00)	*H* = 98.866	< 0.001
Gender				*χ* ^2^ = 9.886	0.0071
1 = Male	123 (6.5%)	202 (6.4%)	74 (9.5%)		
2 = Female	1783 (93.5%)	2950 (93.6%)	707 (90.5%)		
Rural				*χ* ^2^ = 1.783	0.41
1 = Rural	1407 (73.8%)	2298 (72.9%)	557 (71.3%)		
2 = Urban	499 (26.2%)	854 (27.1%)	224 (28.7%)		
LeftBy				*χ* ^2^ = 29.736	< 0.001
1 = Yes	1062 (55.7%)	1570 (49.8%)	352 (45.1%)		
2 = No	844 (44.3%)	1582 (50.2%)	429 (54.9%)		
Religion				*χ* ^2^ = 0.135	0.9348
1 = Yes	109 (5.7%)	179 (5.7%)	47 (6.0%)		
2 = No	1797 (94.3%)	2973 (94.3%)	734 (94.0%)		
Married				*χ* ^2^ = 13.362	0.0096
1 = Single	1773 (93.0%)	2884 (91.5%)	694 (88.9%)		
2 = Married	125 (6.6%)	257 (8.2%)	83 (10.6%)		
3 = Other	8 (0.4%)	11 (0.3%)	4 (0.5%)		
Level				*χ* ^2^ = 3.350	0.1873
1 = Grade 3B	295 (15.5%)	434 (13.8%)	105 (13.4%)		
2 = Grade 3A	1611 (84.5%)	2718 (86.2%)	676 (86.6%)		
Education				*χ* ^2^ = 32.913	< 0.001
1 = Technical secondary school	59 (3.1%)	74 (2.3%)	18 (2.3%)		
2 = Junior college	1314 (68.9%)	2342 (74.3%)	616 (78.9%)		
3 = Bachelor’s degree or above	533 (28.0%)	736 (23.4%)	147 (18.8%)		
Exposure				*χ* ^2^ = 171.665	< 0.001
1 = Yes	1155 (60.6%)	1526 (48.4%)	263 (33.7%)		
2 = No	751 (39.4%)	1626 (51.6%)	518 (66.3%)		
Work Time				*χ* ^2^ = 72.886	< 0.001
1 = Less than 41	400 (20.9%)	874 (27.7%)	272 (34.8%)		
2 = 41–55	1183 (62.1%)	1874 (59.5%)	429 (55.0%)		
3 = 56–70	251 (13.2%)	324 (10.3%)	61 (7.8%)		
4 = More than 70	72 (3.8%)	80 (2.5%)	19 (2.4%)		
Sleep quality			*χ* ^2^ = 352.862	< 0.001	
1 = Self‐perceived good	218 (11.4%)	668 (21.2%)	264 (33.8%)		
2 = Self‐perceived average	1147 (60.2%)	2059 (65.3%)	454 (58.1%)		
3 = Self‐perceived poor	541 (28.4%)	425 (13.5%)	63 (8.1%)		
Alcohol				*χ* ^2^ = 3.366	0.1858
1 = Yes	49 (2.6%)	63 (2.0%)	12 (1.5%)		
2 = No	1857 (97.4%)	3089 (98.0%)	769 (98.5%)		
Smoke				*χ* ^2^ = 0.788	0.6744
1 = Yes	29 (1.5%)	56 (1.8%)	11 (1.4%)		
2 = No	1877 (98.5%)	3096 (98.2%)	770 (98.6%)		
Exercise				*χ* ^2^ = 299.098	< 0.001
1 = Yes	403 (21.1%)	1166 (37.0%)	426 (54.5%)		
2 = No	1503 (78.9%)	1986 (63.0%)	355 (45.5%)		
Chronic disease				*χ* ^2^ = 21.802	< 0.001
1 = Yes	92 (4.8%)	94 (3.0%)	12 (1.5%)		
2 = No	1814 (95.2%)	3058 (97.0%)	769 (98.5%)		

An ordinal logistic regression analysis was performed to further identify factors associated with ordered profile membership. The proportional‐odds assumption was evaluated using a global Brant‐style parallelism test (*χ* ^2^ = 18.508, df = 26, *p* = 0.857), as shown in Table S4. In this model, ORs greater than 1 indicated greater odds of membership in a more resource‐constrained profile. As shown in Figure 2, more frequent night shifts, longer weekly working hours, fair or poor sleep quality, and lack of regular exercise were associated with greater odds of membership in a more resource‐constrained profile. Among these factors, poor sleep quality showed the strongest association (OR = 3.475, 95% CI: 2.883–4.188, *p* < 0.001), followed by lack of regular exercise (OR = 2.241, 95% CI: 2.005–2.506, *p* < 0.001). In contrast, higher individual income, being married, working in a higher‐level hospital, higher educational background, absence of occupational exposure, and absence of chronic disease were associated with lower odds of membership in a more resource‐constrained profile. Detailed ordered and multinomial logistic regression results are reported in Tables S3 and S5. Variables significantly associated with profile membership were retained as covariates for the residualized network sensitivity analyses.

**FIGURE 2:**
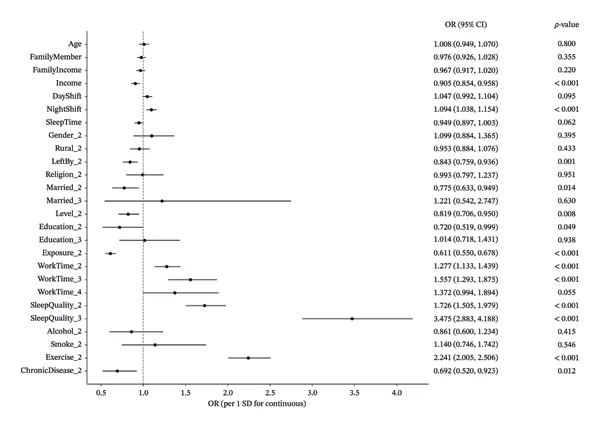
Factors associated with latent profile membership. *Note*. OR, odds ratio; 95% CI, 95% confidence interval.

### 3.3. Network Structure Across Different Groups

#### 3.3.1. Network Plot

Figure 3 presents a comparative visualization of the network structures across the three latent profile groups. All networks were plotted using the same layout to ensure comparability. The primary differences were observed in connectivity patterns and edge weights. The strongest connections tended to cluster within the same psychological domain—either PID, PSR, or ADP—rather than across domains. This pattern indicated a high degree of internal cohesion within each construct.

**FIGURE 3:**
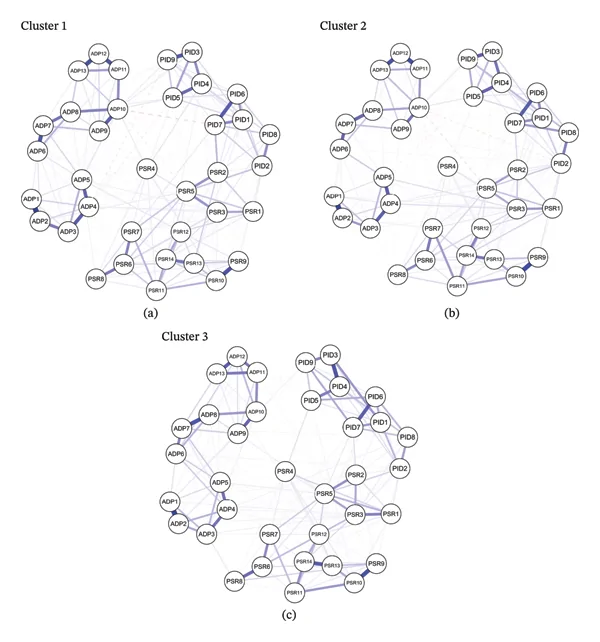
Network structures of the three latent profiles. (a) Resource‐constrained adaptive profile. (b) Moderate resource‐adaptation profile. (c) High resource‐adaptation profile.

Table 4 summarizes the global characteristics of the class‐specific networks. Although all three networks contained the same 36 nodes, their overall connectivity patterns differed. The resource‐constrained adaptive profile had the largest number of nonzero edges (214) and the highest density (0.340), followed by the moderate resource‐adaptation profile (206 nonzero edges; density = 0.327). The high resource‐adaptation profile had fewer nonzero edges (189) and the lowest density (0.300), but it showed the highest global strength (16.223) and the largest mean absolute nonzero edge weight (0.086). This pattern suggests that the high resource‐adaptation profile was characterized by fewer but stronger contemporaneous associations among PID, PSR, and ADP indicators.

**TABLE 4:** Global characteristics of the class‐specific networks.

Class	Nodes	Nonzero edges	Density	Global strength	Mean absolute nonzero edge	Positive edge proportion	Negative edge proportion
1	36	214	0.340	15.854	0.074	0.314	0.025
2	36	206	0.327	14.804	0.072	0.284	0.043
3	36	189	0.300	16.223	0.086	0.284	0.016

Positive edges were more common than negative edges in all three networks. The resource‐constrained adaptive profile showed the highest positive edge proportion (0.314), whereas the moderate resource‐adaptation and high resource‐adaptation profiles had the same positive edge proportion (0.284). Negative edges were relatively infrequent, with the highest negative edge proportion observed in the moderate resource‐adaptation profile (0.043) and the lowest in the high resource‐adaptation profile (0.016). These results indicate that the three profiles differed not only in mean levels of internal resources but also in the density, strength, and valence distribution of their resource‐adaptation networks.

#### 3.3.2. Network Centrality Indices

Figure 4 compares node‐level centrality patterns across the three latent profiles. To facilitate visual comparison, node centrality estimates from each profile‐specific network were displayed in the same graph, with separate lines representing the three profiles. Overall, the centrality patterns showed profile‐specific variation, indicating that the most influential nodes were not uniformly distributed across the three resource‐adaptation profiles.

**FIGURE 4:**
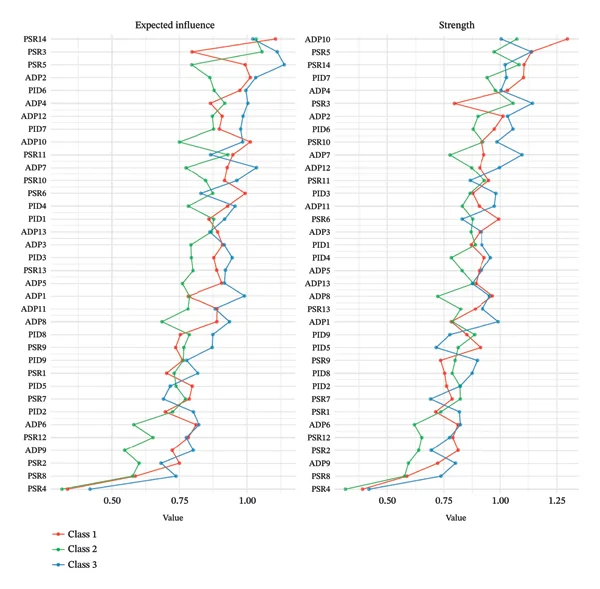
Comparison of node centrality across the three latent profiles.

For node‐level centrality, relatively higher values were mainly observed for nodes reflecting problem‐solving, self‐regulation, calm coping, and adaptive adjustment. These included PSR indicators such as PSR14 (Problem solving), PSR3 (Calm handling), and PSR5 (Control ability), as well as ADP indicators related to emergency response and behavioral regulation, such as ADP4 (Clear thinking) and ADP10 (Behavioral adjustment). Several PID nodes, including PID2 (Social confidence) and PID8 (Social interaction), also showed visible centrality in specific profiles. This pattern indicates that profile‐specific networks were organized not only around resilience‐related capacities but also around interpersonal confidence and adaptive task regulation.

Bridge centrality results are shown in Figure 5, and the top bridge expected influence nodes for each profile are summarized in Table 5. In the resource‐constrained adaptive profile, the leading bridge nodes were PSR2 (Achievement pride), PID8 (Social interaction), PID2 (Social confidence), PSR3 (Calm handling), and PSR1 (Problem solving). In the moderate resource‐adaptation profile, the top bridge nodes were PID2 (Social confidence), PSR3 (Calm handling), PSR2 (Achievement pride), ADP4 (Clear thinking), and PID8 (Social interaction). In the high resource‐adaptation profile, the leading bridge nodes were PID2 (Social confidence), PSR5 (Control ability), PSR3 (Calm handling), PID8 (Social interaction), and PSR2 (Achievement pride).

**FIGURE 5:**
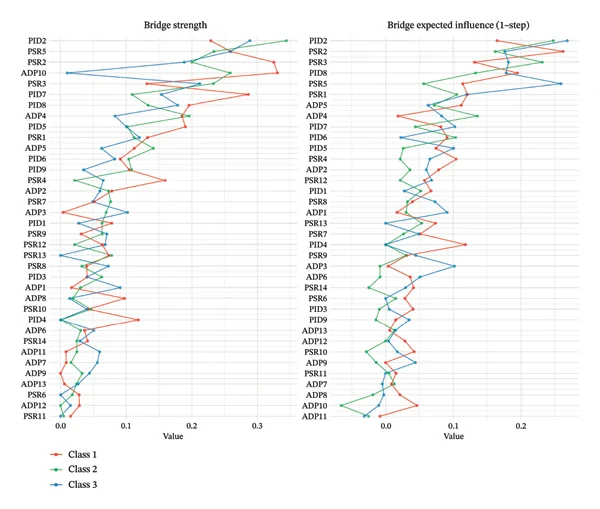
Comparison of bridge centrality across the three latent profiles.

**TABLE 5:** Top bridge expected influence nodes in each latent profile.

Class	Top bridge expected influence nodes
1	PSR2, PID8, PID2, PSR3, PSR1
2	PID2, PSR3, PSR2, ADP4, PID8
3	PID2, PSR5, PSR3, PID8, PSR2

Across the three profiles, PID2 (Social confidence), PID8 (Social interaction), PSR2 (Achievement pride), and PSR3 (Calm handling) repeatedly appeared among the top bridge expected influence nodes. These results suggest that cross‐domain connectivity between PID, PSR, and ADP was primarily organized around interpersonal confidence, social engagement, achievement‐related self‐evaluation, and calm handling of situations. ADP4 (Clear thinking) appeared among the leading bridge nodes only in the moderate resource‐adaptation profile, whereas PSR5 (Control ability) appeared among the leading bridge nodes only in the high resource‐adaptation profile, indicating profile‐specific differences in how adaptive and resilience‐related indicators contributed to cross‐domain associations.

#### 3.3.3. Network Comparison

Network comparison tests (NCTs) were conducted to compare the class‐specific networks in terms of global strength and overall network structure, as shown in Table 6. Consistent with the global network characteristics reported in Table 4, the moderate resource‐adaptation profile showed the lowest global strength (14.804), whereas the high resource‐adaptation profile showed the highest global strength (16.223).

**TABLE 6:** Network comparison test results for class‐specific networks.

Comparison	Global strength difference	Global strength *p*	Maximum edge difference	Network invariance *p*
Class 1 vs. Class 2	1.050	0.002	0.107	0.163
Class 1 vs. Class 3	0.369	0.372	0.176	0.031
Class 2 vs. Class 3	1.419	< 0.001	0.134	0.281

*Note:* Global strength difference refers to the absolute difference in global strength between two networks. Global strength p indicates the permutation‐based significance test for the global strength difference. Maximum edge difference refers to the largest absolute difference in corresponding edge weights between two networks. Network invariance *p* indicates the permutation‐based test of overall network structure invariance. *p* < 0.05 was considered statistically significant.

The comparison between the resource‐constrained adaptive profile and the moderate resource‐adaptation profile showed a significant global strength difference (difference = 1.050, *p* = 0.002), indicating stronger overall connectivity in the resource‐constrained adaptive profile. However, the overall network structure did not differ significantly between these two profiles (network invariance *p* = 0.163). Similarly, the comparison between the moderate resource‐adaptation profile and the high resource‐adaptation profile showed a significant global strength difference (difference = 1.419, *p* < 0.001), with stronger overall connectivity in the high resource‐adaptation profile, whereas the overall network structure was not significantly different (network invariance *p* = 0.281).

In contrast, the comparison between the resource‐constrained adaptive profile and the high resource‐adaptation profile did not show a significant global strength difference (difference = 0.369, *p* = 0.372), but the network invariance test was significant (*p* = 0.031). This finding suggests that these two profiles differed more in the organization of specific associations than in the overall magnitude of connectivity. The maximum edge differences ranged from 0.107 to 0.176 across pairwise comparisons, with the largest value observed between the resource‐constrained adaptive and high resource‐adaptation profiles.

### 3.4. Robustness Analysis

Several robustness analyses were conducted to examine the stability of the regression and network findings. First, regression diagnostics supported the ordered logistic model, with no evidence of violation of the proportional‐odds assumption. A multinomial logistic regression sensitivity analysis showed a broadly consistent pattern of associations, particularly for income, night‐shift frequency, occupational exposure, weekly working hours, sleep quality, and exercise. In addition, the hospital‐cluster robust sensitivity analysis showed that the direction of ORs remained unchanged and that most statistically significant associations were preserved (Table S6).

Second, residualized network models were estimated after adjusting PID, PSR, and ADP items for covariates significantly associated with profile membership. As shown in Figures S1–S3, the residualized networks illustrate patterns broadly consistent with the base networks. Class 2 retained the weakest overall connectivity, whereas Class 3 retained the highest global strength and the largest mean absolute nonzero edge weight (Table S7). Residualized NCTs also supported the main findings: Class 1 versus Class 2 and Class 2 versus Class 3 differed significantly in global strength, whereas Class 1 versus Class 3 showed evidence of network‐structure difference without a significant global‐strength difference (Table S8). The leading bridge expected‐influence nodes are summarized in Table S9 and Figure S4, and complete centrality comparisons are shown in Figures S5 and S6. These results indicate that the main profile‐specific network patterns were not materially altered after covariate adjustment.

Third, bootstrap stability analyses supported the robustness of centrality estimation. In the base networks, the CS coefficients for strength and expected influence were 0.750 across all three profiles. The same coefficients were observed in the residualized networks, indicating that standard centrality stability was preserved after covariate adjustment. Bridge centrality also showed acceptable stability, with CS coefficients ranging from 0.594 to 0.750 across base and residualized networks. Nonparametric bootstrap analyses indicated wider uncertainty around individual edge weights, supporting our decision to emphasize global strength, network invariance, and bridge‐centrality patterns rather than individual edges. Detailed robustness results are provided in Tables S10–S11 and Figures S7–S24.

## 4. Discussion

### 4.1. Principal Findings

This multicenter cross‐sectional study identified three ordered resource‐adaptation profiles among 5839 junior nurses exposed to workplace bullying: the resource‐constrained adaptive profile, the moderate resource‐adaptation profile, and the high resource‐adaptation profile. These profiles differed not only in the mean levels of PID, PSR, and ADP but also in the organization of associations among these resources. The high resource‐adaptation profile showed the highest global strength and the largest mean absolute nonzero edge weight, despite having fewer nonzero edges and lower network density. In contrast, the moderate resource‐adaptation profile showed the weakest overall connectivity. Bridge expected influence analyses further indicated that PID2 (Social confidence), PID8 (Social interaction), PSR2 (Achievement pride), and PSR3 (Calm handling) repeatedly served as cross‐domain connectors between PID, PSR, and ADP. Robustness analyses, including ordered‐model diagnostics, multinomial logistic sensitivity analysis, hospital‐cluster robust adjustment, residualized networks, and bootstrap stability tests, generally supported the stability of the main findings.

Taken together, these findings extend the understanding of junior nurses’ psychological adaptation after workplace bullying by showing that internal resources are not merely high or low in level but are configured and interconnected in profile‐specific ways. This pattern is theoretically compatible with the COR theory, which emphasizes that stress‐related adaptation depends on how individuals conserve, mobilize, and invest available resources under threatening conditions.

### 4.2. Resource‐Adaptation Profiles Among Junior Nurses Exposed to Workplace Bullying

From the perspective of COR theory, workplace bullying can be conceptualized as a resource‐threatening work context in which interpersonal mistreatment may challenge junior nurses’ identity‐related, emotional‐cognitive, and behavioral adaptation resources. In this study, PID represented an identity‐related resource, PSR represented an emotional‐cognitive self‐regulatory resource, and ADP represented a behavioral adaptation resource. The three latent profiles therefore should not be understood as purely empirical categories; rather, they represent different configurations of internal resources among junior nurses facing workplace bullying. This interpretation is consistent with evidence that workplace harassment and nurse bullying are linked to both individual well‐being and organizational functioning, while also reflecting hostile workplace conditions that require personal and organizational responses [37, 38].

The resource‐constrained adaptive profile was characterized by comparatively lower levels of PID, PSR, and ADP. This pattern may reflect a constrained internal resource configuration in which junior nurses retain some capacity for task adaptation but have fewer identity‐related and resilience‐related resources available to support sustained occupational adjustment. Within a COR framework, such a profile may be consistent with limited resource conservation capacity under continuing interpersonal stress. Previous studies of new graduate and novice nurses have similarly shown that workplace bullying is associated with burnout, reduced work productivity, and impaired early‐career functioning [39, 40].

The moderate resource‐adaptation profile, which represented the largest subgroup, showed intermediate levels across all three domains. This profile may represent a state of functional resource maintenance, in which junior nurses maintain adequate professional functioning but do not show the stronger resource integration observed in the high resource‐adaptation profile. This interpretation is important because moderate resource levels do not necessarily indicate a stable or optimal resource system. Rather, these nurses may occupy an intermediate position in which additional contextual demands, such as shift burden or poor recovery conditions, could be associated with movement toward a more constrained configuration, whereas adequate support may be associated with stronger resource consolidation. This interpretation is also consistent with evidence that burnout and fatigue among nurses are shaped by workload, shift work, recovery opportunities, and organizational conditions [41, 42].

The high resource‐adaptation profile showed the highest mean scores across PID, PSR, and ADP. From the COR perspective, this pattern may reflect stronger resource conservation and resource mobilization, including a more coherent sense of professional value, greater self‐regulatory capacity, and more effective behavioral adaptation. This interpretation aligns with evidence that nurse resilience is a dynamic process supported by personal and contextual resources and that ADP involves adjusting effectively to changing task, interpersonal, and organizational demands [43, 44].

### 4.3. Network Organization, Bridge Nodes, and Resource Trade‐Offs

The integration of LPA and network analysis provided complementary information. LPA identified person‐centered heterogeneity in internal resource levels, whereas network analysis clarified how PID, PSR, and ADP were organized within each profile. This distinction is central to the contribution of the study: nurses in different profiles differed not only in how many internal resources they reported but also in how strongly and selectively these resources were connected [45, 46].

The high resource‐adaptation profile had fewer nonzero edges and lower density than the other two profiles, yet it showed the highest global strength and the largest mean absolute nonzero edge weight. This pattern suggests a more selective but stronger organization of internal resources. In COR terms, this may be compatible with more efficient resource integration, in which fewer but stronger connections support coordination across identity‐related, resilience‐related, and adaptive domains [47]. Conversely, the moderate resource‐adaptation profile showed the lowest global strength, suggesting that intermediate mean resource levels may coexist with weaker overall resource integration. The resource‐constrained adaptive profile showed relatively high density but lower mean edge strength than the high resource‐adaptation profile, which may indicate a broader but less selectively consolidated association pattern.

NCTs further supported this interpretation. The resource‐constrained adaptive and moderate resource‐adaptation profiles differed significantly in global strength, as did the moderate and high resource‐adaptation profiles. By contrast, the resource‐constrained adaptive and high resource‐adaptation profiles did not differ significantly in global strength but showed evidence of network‐structure difference. This suggests that the distinction between profiles cannot be reduced to overall connectivity alone; the organization of specific associations also matters [48].

Bridge centrality results highlighted several cross‐domain connectors. Across profiles, PID2 (Social confidence), PID8 (Social interaction), PSR2 (Achievement pride), and PSR3 (Calm handling) repeatedly appeared among the leading bridge expected influence nodes. These nodes may represent points at which identity‐related resources, resilience‐related resources, and ADP are linked [49]. PID2 (Social confidence) and PID8 (Social interaction) indicate the interpersonal component of PID, suggesting that confidence in social engagement and willingness to interact may help connect professional self‐perception with resilience and adaptive functioning. PSR2 (Achievement pride) reflects achievement‐related self‐evaluation, whereas PSR3 (Calm handling) reflects emotional and cognitive regulation under challenging conditions. Their repeated bridge roles suggest that resource integration among junior nurses exposed to workplace bullying may rely partly on interpersonal confidence, social engagement, achievement‐related meaning, and calm coping.

Profile‐specific bridge patterns also provided additional insight. ADP4 (Clear thinking) appeared among the leading bridge nodes only in the moderate resource‐adaptation profile, suggesting that cognitive organization during urgent situations may be particularly relevant when resources are present but not highly integrated [50]. PSR5 (Control ability) appeared among the leading bridge nodes only in the high resource‐adaptation profile, suggesting that perceived control may become more central to cross‐domain resource coordination when the overall resource configuration is more favorable. These interpretations should be regarded as hypothesis‐generating and require confirmation in longitudinal or intervention studies.

The negative edges observed in the networks were relatively infrequent but theoretically meaningful. The moderate resource‐adaptation profile showed the highest negative edge proportion, whereas the high resource‐adaptation profile showed the lowest. From a resource‐oriented perspective, negative associations may be compatible with potential resource trade‐offs, in which investment in one adaptive function is associated with lower activation of another resource under certain conditions. For example, behavioral adjustment or interpersonal adaptation may require self‐regulatory effort that is not always aligned with perceived control or achievement‐related self‐evaluation. However, the bootstrap analyses indicated that individual edge weights should be interpreted cautiously. Therefore, these negative associations should be treated as preliminary indicators of possible resource tensions rather than evidence of stable causal trade‐off mechanisms [51].

### 4.4. Contextual Factors Associated With Profile Membership

The regression findings suggest that latent profile membership was associated with several contextual resource conditions. More frequent night shifts, longer weekly working hours, poorer sleep quality, and lack of regular exercise were associated with greater odds of membership in a more resource‐constrained profile. These factors can be interpreted as workload‐related demands and recovery‐related constraints. Recent nursing research has shown that shift work and night‐shift burden are closely linked to sleep disturbance and poorer recovery, while reviews of nurses’ mental health have also identified shift work, job demands, and insufficient job resources as important contextual risks [52]. From the COR perspective, repeated shift burden and insufficient recovery may be associated with reduced capacity to conserve or replenish internal resources, especially among junior nurses who are still developing professional confidence and workplace adaptation skills.

In contrast, higher individual income, being married, working in a higher‐level hospital, higher educational background, absence of occupational exposure, and absence of chronic disease were associated with lower odds of membership in a more resource‐constrained profile. These variables may reflect socioeconomic, interpersonal, institutional, and health‐related resource conditions. For example, higher income and educational background may indicate greater access to material and developmental resources; marital support may provide emotional resources; higher‐level hospitals may offer more structured training and organizational support; and absence of occupational exposure or chronic disease may indicate fewer additional resource burdens. Recent studies also suggest that perceived social support, PID, organizational support, and resilience are closely linked to nurses’ psychological functioning and intention to stay [50, 53]. Occupational exposure and work‐related injury may add further psychological and safety‐related burdens, particularly when combined with shift‐related sleep disruption or reduced job control [54]. These associations should not be interpreted as causal predictors, but they help contextualize how internal resource profiles may be embedded within broader working and living conditions.

The robustness analyses support a cautious interpretation of these contextual findings. The ordered logistic model satisfied the proportional‐odds assumption, and the multinomial logistic sensitivity analysis showed broadly consistent directions of association. After hospital‐cluster robust adjustment, the direction of ORs remained unchanged, and most statistically significant associations were preserved, although some associations, such as sleep time and hospital level, became nonsignificant. These findings suggest that the main contextual patterns were generally stable, while also indicating that hospital‐level clustering and measurement variation should be considered when interpreting institutional and recovery‐related factors. This caution is consistent with recent multicenter nursing research showing that nurses are often nested within institutional environments, where staffing structures, work environments, and organizational practices may shape workforce outcomes [55, 56]. Therefore, contextual associations should be interpreted as profile‐related correlates within a clustered cross‐sectional design rather than as evidence of individual‐ or hospital‐level causal effects [57].

### 4.5. Practical Implications

The findings have several implications for nursing management. First, subgroup identification may help nurse managers recognize that junior nurses exposed to workplace bullying are not psychologically homogeneous. Nurses in the resource‐constrained adaptive profile may require earlier and more structured support aimed at reducing resource depletion and strengthening basic recovery conditions. Such support may include workload review, more careful night‐shift scheduling, postexposure support after occupational incidents, sleep and recovery promotion, and accessible psychological consultation.

Second, nurses in the moderate resource‐adaptation profile should not be overlooked. Although this was the largest group and showed intermediate levels of internal resources, it also had the weakest global network strength. This suggests that moderate resource levels may not necessarily correspond to strong resource integration. Managerial strategies for this group may focus on consolidating existing resources through mentoring, peer support, reflective practice, and structured feedback that reinforces professional confidence and adaptive problem handling. Formal transition programs and structured mentorship have been shown to support new nurses’ confidence, clinical competence, communication, and retention, which makes them particularly suitable for nurses whose resources are present but weakly integrated [58]. Peer‐based and reflective approaches may also help normalize help‐seeking, strengthen workplace belonging, and provide opportunities to rehearse adaptive responses to difficult interpersonal or clinical situations [59].

Third, the high resource‐adaptation profile may provide useful information about favorable resource organization. These nurses showed stronger overall network connectivity despite fewer retained edges, suggesting a more selective and integrated resource structure. However, they should not simply be viewed as self‐sufficient. Sustaining their resources may require opportunities for professional development, recognition, and appropriate participation in peer‐support roles without creating additional role overload.

Finally, bridge nodes such as PID2 (Social confidence), PID8 (Social interaction), PSR2 (Achievement pride), and PSR3 (Calm handling) may inform future resource‐oriented intervention design. Programs that strengthen interpersonal confidence, promote constructive social interaction, recognize professional achievements, and support calm coping under pressure may be particularly relevant. For example, interventions could combine communication‐skills training, structured peer interaction, recognition of professional contribution, and stress‐management exercises rather than focusing on a single resource domain [60]. Nevertheless, because the present study used cross‐sectional data, these nodes should be regarded as hypothesis‐generating indicators rather than confirmed intervention targets. Future longitudinal and intervention studies are needed to test whether modifying these domains can produce sustained improvements in resource configuration and occupational adaptation [61, 62].

### 4.6. Strengths and Limitations

This study has several strengths. First, it focused on a large multicenter sample of junior nurses exposed to workplace bullying, a group that is particularly relevant to workforce stability and early‐career professional development. Second, by combining LPA with network analysis, the study integrated person‐centered and variable‐centered approaches, allowing resource heterogeneity and resource organization to be examined simultaneously. Third, the study strengthened methodological transparency by reporting ordered‐model diagnostics, multinomial logistic sensitivity analysis, hospital‐cluster robust adjustment, residualized network models, NCTs, and bootstrap stability evidence. The CS coefficients for strength and expected influence were stable across both base and residualized networks, supporting the reliability of the centrality patterns reported in this study.

Several limitations should also be acknowledged. First, the cross‐sectional design precludes conclusions about temporal ordering, causal pathways, or resource gain and loss spirals. Therefore, the COR‐informed interpretations should be understood as theoretically plausible explanations rather than evidence of dynamic resource processes. Second, all measures were based on self‐report questionnaires, which may introduce common method variance and response bias, although procedural safeguards and Harman’s single‐factor test suggested that common method bias was not a severe concern. In addition, five‐point Likert‐type items were treated as approximately continuous, and ADP was assessed using selected dimensions rather than the full multidimensional construct. Finally, the generalizability and institutional interpretation of the findings are limited. The sample was drawn from tertiary hospitals in Sichuan Province, and the hospital‐cluster robust analysis was conducted in a slightly reduced sample after cleaning hospital identifiers.

## 5. Conclusion

This study identified three ordered resource‐adaptation profiles among junior nurses exposed to workplace bullying and showed that these profiles differed not only in the mean levels of PID, PSR, and ADP but also in the structural organization of these resources. The high resource‐adaptation profile was characterized by stronger overall network connectivity and more selective retained associations, whereas the moderate resource‐adaptation profile showed the weakest global strength. Bridge nodes including PID2 (Social confidence), PID8 (Social interaction), PSR2 (Achievement pride), and PSR3 (Calm handling) may represent important cross‐domain connectors among identity‐related, resilience‐related, and adaptive resources.

By integrating LPA with network analysis, this study contributes to a more nuanced COR‐informed understanding of how internal resources may be configured and interconnected among junior nurses facing workplace bullying. Methodologically, the findings demonstrate the value of combining person‐centered and network approaches to examine both resource heterogeneity and resource organization. Practically, the results support tailored managerial strategies that address workload demands, recovery conditions, professional confidence, social interaction, achievement recognition, and calm coping. Given the cross‐sectional and self‐report design, these findings should be interpreted cautiously and regarded as a basis for future longitudinal and intervention research rather than evidence of causal resource gain or loss processes.

## Funding

This research was funded by the Humanities and Social Science Fund of Ministry of Education of China (No. 23YJC630130), the Scientific research and development plan of the Affiliated Hospital of North Sichuan Medical College (2023PTRJ001), and the development plan of North Sichuan Medical College (CBY23‐ZDB01).

## Disclosure

The authors declare that the funding sources (Humanities and Social Science Fund of Ministry of Education of China, Scientific Research and Development Plan of the Affiliated Hospital of North Sichuan Medical College, and Development Plan of North Sichuan Medical College) had no involvement in study design, data collection, analysis, interpretation, or manuscript preparation.

## Ethics Statement

This study was approved by the Ethics Committee of the Affiliated Hospital of North Sichuan Medical College (2021ER006‐1).

## Conflicts of Interest

The authors declare no conflicts of interest.

## Supporting Information

Additional supporting information can be found online in the Supporting Information section.

## Supporting information


**Supporting Information** The Supporting Information file presents the questionnaire and variable definitions used in the study (Table S1), classification diagnostics for the selected three‐class latent profile solution (Table S2), ordered and multinomial logistic regression results for factors associated with latent profile membership (Tables S3 and S5), and robustness analyses including proportional‐odds diagnostics (Table S4), hospital‐cluster robust sensitivity analysis (Table S6), residualized network models and network comparison tests (Figures S1–S6 and Tables S7–S9), and bootstrap stability results (Tables S10–S11 and Figures S7–S24).

## Data Availability

The analytic code is available at https://github.com/llanjun/workplace-bullying-lpa-network. The data supporting the findings of this study are not publicly available due to privacy and ethical restrictions. They may be obtained from the corresponding author upon reasonable request, subject to applicable ethical and institutional requirements.
