# Supplementary material for: Professional Identity, Psychological Resilience, and Adaptive Performance in Junior Nurses Exposed to Workplace Bullying: A Latent Profile and Network Analysis
Source: J Nurs Manag. 2026 Oct 29;2026:6681049. doi: 10.1155/jonm/6681049 (PMC13720351; doi:10.1155/jonm/6681049)
Supplement: Supplementary file 1 [file JONM-2026-6681049-s001.docx]

**Supporting Information**

**Abbreviations.** Class 1 = resource-constrained adaptive profile; Class 2 = moderate resource-adaptation profile; Class 3 = high resource-adaptation profile; PID = professional identity; PSR = psychological resilience; ADP = adaptive performance; NCT = Network Comparison Test; CS = correlation-stability coefficient.

# S1. Variables and questionnaire items

This section defines all demographic variables and item-level indicators used in the regression, LPA, and network analyses.

**Supplementary Table S1. Questionnaire variables, response options, and brief item meanings used in the analyses**

| **Variable Name** | **Survey Item** | **Response Options** | **Brief** |
| --- | --- | --- | --- |
| Demographic | | | |
| Gender | Gender | 1=Male, 2=Female | NA |
| Age | Your actual age (years) | Fill in the blank | NA |
| Rural | Your household registration location | 1=Rural, 2=Urban | NA |
| LeftBy | Left-behind experience (defined as having one or both parents working away from home before age 16) | 1=Yes, 2=No | NA |
| Religion | Religious belief | 1=Yes, 2=No | NA |
| Married | Marital status | 1=Single, 2=Married, 3=Other | NA |
| FamilyMember | Number of permanent residents in household | Fill in the blank | NA |
| Level | Training hospital level | 1=Grade 3B, 2=Grade 3A | NA |
| Education | Educational background | 1=Technical secondary school, 2=Junior college, 3=Bachelor's degree or above | NA |
| Exposure | Occupational exposure experience (such as needlestick injuries or radiation exposure) | 1=Yes, 2=No | NA |
| WorkTime | Weekly working hours (hours/week) | 1=Less than 41, 2=41-55, 3=56-70, 4=More than 70 | NA |
| FamilyIncome | Monthly household income per capita (yuan) | Fill in the blank | NA |
| Income | Monthly average salary income (yuan) | Fill in the blank | NA |
| DayShift | Weekly day shift frequency | Fill in the blank | NA |
| NightShift | Weekly night shift frequency | Fill in the blank | NA |
| SleepTime | Daily average sleep time (hours) | Fill in the blank | NA |
| SleepQuality | Sleep quality | 1=Self-perceived good, 2=Self-perceived average, 3=Self-perceived poor | NA |
| Alcohol | Alcohol consumption (standard: drinking liquor or wine more than 50ml at once or beer more than 200ml, drinking at least twice a week for more than 6 consecutive months. Drinking small amounts during holidays does not count) | 1=Yes, 2=No | NA |
| Smoke | Smoking (standard: smoking at least one cigarette per day for more than 6 consecutive months) | 1=Yes, 2=No | NA |
| Exercise | Physical exercise participation (defined as planned and repetitive physical activities) | 1=Yes, 2=No | NA |
| ChronicDisease | Diagnosed chronic diseases in the past year (non-infectious diseases with long-term accumulation causing structural damage, such as cardiovascular diseases) | 1=Yes, 2=No | NA |
| Professional Identity | | | |
| PID1 | Nursing work gives me a sense of value | 1=Strongly disagree, 2=Disagree, 3=Sometimes agree, 4=Agree, 5=Strongly agree | Value sense |
| PID2 | I feel confident in approaching and dealing with anyone I encounter | Same as PID1 | Social confidence |
| PID3 | Understanding and support from colleagues brings me happiness | Same as PID1 | Colleague support |
| PID4 | I firmly believe that people have subjective choice and agency, not only adapting to the work environment but also creating and optimizing it | Same as PID1 | Work agency |
| PID5 | I believe career choice has both necessity and chance, and you can only achieve if you recognize it | Same as PID1 | Career choice |
| PID6 | Nursing work matches my interests and personality | Same as PID1 | Work match |
| PID7 | The nursing profession helps me gain respect from society | Same as PID1 | Social respect |
| PID8 | I enjoy communicating with people and actively seek social contact opportunities | Same as PID1 | Social interaction |
| PID9 | Recognition from doctors and managers brings me happiness | Same as PID1 | Authority recognition |
| Workplace Bullying | | | |
| ABU1 | Being asked to do work outside job scope, such as running errands | 1=Rarely occurs, 2=Seldom occurs, 3=Sometimes, 4=Often occurs, 5=Frequently occurs | NA |
| ABU2 | Receiving closer supervision than other colleagues | Same as ABU1 | NA |
| ABU3 | Experiencing excessive teasing, insults, or offense from colleagues or supervisors | Same as ABU1 | NA |
| ABU4 | Having to work twice as hard as other colleagues | Same as ABU1 | NA |
| ABU5 | Supervisors undervalue my work and efforts | Same as ABU1 | NA |
| ABU6 | Receiving unfair humiliation at work | Same as ABU1 | NA |
| Psychological Resilience | | | |
| PSR1 | I usually have multiple ways to handle things | 1=Never, 2=Rarely, 3=Sometimes, 4=Often, 5=Always | Problem solving |
| PSR2 | I am proud of my current achievements | Same as PSR1 | Achievement pride |
| PSR3 | I usually handle situations calmly | Same as PSR1 | Calm handling |
| PSR4 | I am my own friend | Same as PSR1 | Self friendship |
| PSR5 | I feel I can manage many things well | Same as PSR1 | Control ability |
| PSR6 | I have strong willpower | Same as PSR1 | Strong will |
| PSR7 | Nothing can defeat me | Same as PSR1 | Invincibility |
| PSR8 | I can control myself | Same as PSR1 | Self control |
| PSR9 | I am interested in many things in life | Same as PSR1 | Life interest |
| PSR10 | I often find things that make me happy | Same as PSR1 | Happiness finding |
| PSR11 | I believe I can overcome any adversity | Same as PSR1 | Adversity overcoming |
| PSR12 | In emergencies, I am someone others can rely on | Same as PSR1 | Emergency reliability |
| PSR13 | My life is meaningful | Same as PSR1 | Life meaning |
| PSR14 | When I encounter difficulties, I can find ways to get out of trouble | Same as PSR1 | Problem solving |
| Adaptive Performance | | | |
| ADP1 | I can remain calm when work arrangements are too tight | 1=Completely disagree, 2=Somewhat disagree, 3=Uncertain, 4=Somewhat agree, 5=Completely agree | Tight schedule calmness |
| ADP2 | I can remain calm when work pressure is too high | Same as ADP1 | Pressure calmness |
| ADP3 | I can control my emotions when dealing with urgent problems | Same as ADP1 | Emotional control |
| ADP4 | When dealing with urgent problems, I usually think clearly and prioritize | Same as ADP1 | Clear thinking |
| ADP5 | I can handle urgent problems objectively | Same as ADP1 | Objective handling |
| ADP6 | I can work well with people of different personalities | Same as ADP1 | Personality adaptation |
| ADP7 | I can integrate into different values, customs, and cultures | Same as ADP1 | Cultural integration |
| ADP8 | I am willing to maintain good relationships with people from different cultural backgrounds | Same as ADP1 | Intercultural harmony |
| ADP9 | I am willing to modify my behavior/appearance to adapt to other cultures and customs | Same as ADP1 | Behavioral modification |
| ADP10 | I will adjust my behavior when necessary | Same as ADP1 | Behavioral adjustment |
| ADP11 | I actively understand the work atmosphere, needs, and values of other departments | Same as ADP1 | Departmental understanding |
| ADP12 | I actively understand the hospital's organizational atmosphere, development direction, and values | Same as ADP1 | Organizational understanding |
| ADP13 | I understand the meaning of behaviors in other cultures and make corresponding adjustments | Same as ADP1 | Cultural adjustment |

**Note.** Brief item meanings provide concise node labels for interpreting the network and bridge-centrality results. For scale items with identical response options, “Same as” indicates that the response scale follows the first item in that scale block.

# S2. LPA classification results

The main manuscript reports LPA model-fit indices and profile-level mean scores. The supplementary LPA result below focuses on classification uncertainty for the selected three-class solution.

**Supplementary Table S2. Average posterior probability matrix for the selected three-class LPA model**

| **Assigned class** | **Class 1** | **Class 2** | **Class 3** |
| --- | --- | --- | --- |
| Class 1 | 0.975 | 0.025 | 0.000 |
| Class 2 | 0.013 | 0.981 | 0.006 |
| Class 3 | 0.000 | 0.029 | 0.971 |

**Note.** Rows indicate assigned class and columns indicate the mean posterior probability of membership in each latent class. Diagonal values ranged from 0.971 to 0.981, indicating clear class separation and low classification uncertainty.

# S3. Logistic regression and model diagnostics

Ordered logistic regression was the primary model because the three latent profiles followed an ordered resource-adaptation gradient. Multinomial logistic regression and hospital-cluster robust standard errors were used as sensitivity analyses.

**Supplementary Table S3. Ordered logistic regression predicting latent profile membership**

| **Variable** | **Coef** | **SE** | **Wald chi-square** | **OR (95% CI)** | **p** |
| --- | --- | --- | --- | --- | --- |
| Age | 0.008 | 0.030 | 0.064 | 1.008 (0.949, 1.070) | 0.800 |
| FamilyMember | -0.025 | 0.027 | 0.854 | 0.976 (0.926, 1.028) | 0.355 |
| FamilyIncome | -0.033 | 0.027 | 1.505 | 0.967 (0.917, 1.020) | 0.220 |
| Income | -0.100 | 0.029 | 11.726 | 0.905 (0.854, 0.958) | <0.001 |
| DayShift | 0.045 | 0.027 | 2.794 | 1.047 (0.992, 1.104) | 0.095 |
| NightShift | 0.090 | 0.027 | 11.058 | 1.094 (1.038, 1.154) | <0.001 |
| SleepTime | -0.053 | 0.028 | 3.482 | 0.949 (0.897, 1.003) | 0.062 |
| Gender_2 | 0.094 | 0.111 | 0.723 | 1.099 (0.884, 1.365) | 0.395 |
| Rural_2 | -0.049 | 0.062 | 0.616 | 0.953 (0.844, 1.076) | 0.433 |
| LeftBy_2 | -0.171 | 0.054 | 10.178 | 0.843 (0.759, 0.936) | 0.001 |
| Religion_2 | -0.007 | 0.112 | 0.004 | 0.993 (0.797, 1.237) | 0.951 |
| Married_2 | -0.255 | 0.103 | 6.068 | 0.775 (0.633, 0.949) | 0.014 |
| Married_3 | 0.199 | 0.414 | 0.232 | 1.221 (0.542, 2.747) | 0.630 |
| Level_2 | -0.199 | 0.076 | 6.927 | 0.819 (0.706, 0.950) | 0.008 |
| Education_2 | -0.329 | 0.167 | 3.865 | 0.720 (0.519, 0.999) | 0.049 |
| Education_3 | 0.014 | 0.176 | 0.006 | 1.014 (0.718, 1.431) | 0.938 |
| Exposure_2 | -0.493 | 0.053 | 85.031 | 0.611 (0.550, 0.678) | <0.001 |
| WorkTime_2 | 0.244 | 0.061 | 15.980 | 1.277 (1.133, 1.439) | <0.001 |
| WorkTime_3 | 0.443 | 0.095 | 21.814 | 1.557 (1.293, 1.875) | <0.001 |
| WorkTime_4 | 0.316 | 0.165 | 3.696 | 1.372 (0.994, 1.894) | 0.055 |
| SleepQuality_2 | 0.546 | 0.070 | 61.048 | 1.726 (1.505, 1.979) | <0.001 |
| SleepQuality_3 | 1.245 | 0.095 | 170.903 | 3.475 (2.883, 4.188) | <0.001 |
| Alcohol_2 | -0.150 | 0.184 | 0.665 | 0.861 (0.600, 1.234) | 0.415 |
| Smoke_2 | 0.131 | 0.216 | 0.365 | 1.140 (0.746, 1.742) | 0.546 |
| Exercise_2 | 0.807 | 0.057 | 200.923 | 2.241 (2.005, 2.506) | <0.001 |
| ChronicDisease_2 | -0.367 | 0.147 | 6.287 | 0.692 (0.520, 0.923) | 0.012 |

**Note.** Odds ratios greater than 1 indicate higher odds of membership in a more resource-constrained profile. The largest positive associations were observed for poor sleep quality and lack of regular exercise, whereas higher income and absence of occupational exposure were associated with lower odds of membership in the more resource-constrained profile.

**Supplementary Table S4. Brant-style parallelism test for the ordered logistic model**

| **Test** | **Chi-square** | **df** | **p** |
| --- | --- | --- | --- |
| Global parallelism test | 18.508 | 26 | 0.857 |
| Age | 0.157 | 1 | 0.692 |
| FamilyMember | 0.296 | 1 | 0.586 |
| FamilyIncome | 0.276 | 1 | 0.600 |
| Income | 0.128 | 1 | 0.721 |
| DayShift | 0.150 | 1 | 0.699 |
| NightShift | 0.607 | 1 | 0.436 |
| SleepTime | 1.052 | 1 | 0.305 |
| Gender_2 | 6.921 | 1 | 0.009 |
| Rural_2 | 0.238 | 1 | 0.626 |
| LeftBy_2 | 0.340 | 1 | 0.560 |
| Religion_2 | 0.062 | 1 | 0.804 |
| Married_2 | 0.065 | 1 | 0.799 |
| Married_3 | 0.423 | 1 | 0.515 |
| Level_2 | 0.415 | 1 | 0.519 |
| Education_2 | 0.333 | 1 | 0.564 |
| Education_3 | 0.493 | 1 | 0.482 |
| Exposure_2 | 2.503 | 1 | 0.114 |
| WorkTime_2 | 0.002 | 1 | 0.968 |
| WorkTime_3 | 0.250 | 1 | 0.617 |
| WorkTime_4 | 0.521 | 1 | 0.470 |
| SleepQuality_2 | 0.280 | 1 | 0.597 |
| SleepQuality_3 | 0.298 | 1 | 0.585 |
| Alcohol_2 | 0.041 | 1 | 0.840 |
| Smoke_2 | 2.135 | 1 | 0.144 |
| Exercise_2 | 0.000 | 1 | 0.982 |
| ChronicDisease_2 | 0.630 | 1 | 0.428 |

**Note.** The global test was not significant (chi-square = 18.508, df = 26, p = 0.857), supporting the proportional-odds assumption for the primary ordered logistic model. Individual tests are provided for transparency.

**Supplementary Table S5. Multinomial logistic regression predicting latent profile membership**

| **Comparison** | **Variable** | **Coef** | **SE** | **Wald chi-square** | **OR (95% CI)** | **p** |
| --- | --- | --- | --- | --- | --- | --- |
| Cluster 2 vs Cluster 1 | const | 0.803 | 0.409 | 3.847 | 2.232 (1.001, 4.978) | 0.050 |
| Cluster 2 vs Cluster 1 | Age | 0.001 | 0.036 | 0.000 | 1.001 (0.933, 1.074) | 0.983 |
| Cluster 2 vs Cluster 1 | FamilyMember | 0.009 | 0.031 | 0.078 | 1.009 (0.949, 1.072) | 0.780 |
| Cluster 2 vs Cluster 1 | FamilyIncome | 0.016 | 0.033 | 0.250 | 1.017 (0.953, 1.084) | 0.617 |
| Cluster 2 vs Cluster 1 | Income | 0.076 | 0.034 | 4.926 | 1.079 (1.009, 1.154) | 0.026 |
| Cluster 2 vs Cluster 1 | DayShift | -0.032 | 0.031 | 1.044 | 0.968 (0.911, 1.030) | 0.307 |
| Cluster 2 vs Cluster 1 | NightShift | -0.094 | 0.031 | 9.378 | 0.910 (0.857, 0.967) | 0.002 |
| Cluster 2 vs Cluster 1 | SleepTime | 0.066 | 0.033 | 4.048 | 1.069 (1.002, 1.140) | 0.044 |
| Cluster 2 vs Cluster 1 | Gender_2 | 0.154 | 0.131 | 1.393 | 1.167 (0.903, 1.507) | 0.238 |
| Cluster 2 vs Cluster 1 | Rural_2 | 0.022 | 0.073 | 0.088 | 1.022 (0.886, 1.178) | 0.767 |
| Cluster 2 vs Cluster 1 | LeftBy_2 | 0.181 | 0.062 | 8.380 | 1.198 (1.060, 1.354) | 0.004 |
| Cluster 2 vs Cluster 1 | Religion_2 | 0.020 | 0.131 | 0.023 | 1.020 (0.789, 1.319) | 0.879 |
| Cluster 2 vs Cluster 1 | Married_2 | 0.222 | 0.126 | 3.087 | 1.248 (0.975, 1.598) | 0.079 |
| Cluster 2 vs Cluster 1 | Married_3 | -0.378 | 0.487 | 0.602 | 0.685 (0.264, 1.781) | 0.438 |
| Cluster 2 vs Cluster 1 | Level_2 | 0.212 | 0.087 | 5.934 | 1.237 (1.042, 1.467) | 0.015 |
| Cluster 2 vs Cluster 1 | Education_2 | 0.348 | 0.187 | 3.464 | 1.416 (0.982, 2.042) | 0.063 |
| Cluster 2 vs Cluster 1 | Education_3 | 0.056 | 0.197 | 0.081 | 1.058 (0.719, 1.555) | 0.776 |
| Cluster 2 vs Cluster 1 | Exposure_2 | 0.372 | 0.062 | 36.096 | 1.450 (1.284, 1.637) | <0.001 |
| Cluster 2 vs Cluster 1 | WorkTime_2 | -0.203 | 0.074 | 7.546 | 0.816 (0.706, 0.944) | 0.006 |
| Cluster 2 vs Cluster 1 | WorkTime_3 | -0.357 | 0.109 | 10.664 | 0.700 (0.565, 0.867) | 0.001 |
| Cluster 2 vs Cluster 1 | WorkTime_4 | -0.348 | 0.183 | 3.613 | 0.706 (0.493, 1.011) | 0.057 |
| Cluster 2 vs Cluster 1 | SleepQuality_2 | -0.427 | 0.090 | 22.435 | 0.652 (0.547, 0.779) | <0.001 |
| Cluster 2 vs Cluster 1 | SleepQuality_3 | -1.113 | 0.111 | 99.685 | 0.329 (0.264, 0.409) | <0.001 |
| Cluster 2 vs Cluster 1 | Alcohol_2 | 0.123 | 0.208 | 0.353 | 1.131 (0.753, 1.699) | 0.553 |
| Cluster 2 vs Cluster 1 | Smoke_2 | -0.396 | 0.256 | 2.396 | 0.673 (0.408, 1.111) | 0.122 |
| Cluster 2 vs Cluster 1 | Exercise_2 | -0.703 | 0.069 | 102.275 | 0.495 (0.432, 0.567) | <0.001 |
| Cluster 2 vs Cluster 1 | ChronicDisease_2 | 0.282 | 0.157 | 3.226 | 1.326 (0.975, 1.805) | 0.072 |
| Cluster 3 vs Cluster 1 | const | -0.824 | 0.673 | 1.498 | 0.439 (0.117, 1.641) | 0.221 |
| Cluster 3 vs Cluster 1 | Age | -0.023 | 0.053 | 0.198 | 0.977 (0.881, 1.083) | 0.656 |
| Cluster 3 vs Cluster 1 | FamilyMember | 0.045 | 0.047 | 0.937 | 1.046 (0.955, 1.147) | 0.333 |
| Cluster 3 vs Cluster 1 | FamilyIncome | 0.058 | 0.044 | 1.747 | 1.059 (0.973, 1.154) | 0.186 |
| Cluster 3 vs Cluster 1 | Income | 0.160 | 0.049 | 10.688 | 1.174 (1.066, 1.292) | 0.001 |
| Cluster 3 vs Cluster 1 | DayShift | -0.081 | 0.048 | 2.810 | 0.922 (0.839, 1.014) | 0.094 |
| Cluster 3 vs Cluster 1 | NightShift | -0.126 | 0.049 | 6.523 | 0.882 (0.801, 0.971) | 0.011 |
| Cluster 3 vs Cluster 1 | SleepTime | 0.066 | 0.050 | 1.741 | 1.068 (0.969, 1.177) | 0.187 |
| Cluster 3 vs Cluster 1 | Gender_2 | -0.251 | 0.175 | 2.056 | 0.778 (0.552, 1.097) | 0.152 |
| Cluster 3 vs Cluster 1 | Rural_2 | 0.098 | 0.107 | 0.830 | 1.103 (0.893, 1.361) | 0.362 |
| Cluster 3 vs Cluster 1 | LeftBy_2 | 0.271 | 0.094 | 8.413 | 1.312 (1.092, 1.576) | 0.004 |
| Cluster 3 vs Cluster 1 | Religion_2 | -0.012 | 0.193 | 0.004 | 0.988 (0.677, 1.441) | 0.949 |
| Cluster 3 vs Cluster 1 | Married_2 | 0.478 | 0.174 | 7.564 | 1.614 (1.147, 2.269) | 0.006 |
| Cluster 3 vs Cluster 1 | Married_3 | -0.157 | 0.663 | 0.056 | 0.855 (0.233, 3.133) | 0.813 |
| Cluster 3 vs Cluster 1 | Level_2 | 0.291 | 0.133 | 4.771 | 1.337 (1.030, 1.736) | 0.029 |
| Cluster 3 vs Cluster 1 | Education_2 | 0.452 | 0.291 | 2.403 | 1.571 (0.887, 2.781) | 0.121 |
| Cluster 3 vs Cluster 1 | Education_3 | -0.139 | 0.309 | 0.201 | 0.871 (0.475, 1.595) | 0.654 |
| Cluster 3 vs Cluster 1 | Exposure_2 | 0.856 | 0.095 | 81.788 | 2.354 (1.955, 2.833) | <0.001 |
| Cluster 3 vs Cluster 1 | WorkTime_2 | -0.393 | 0.104 | 14.313 | 0.675 (0.550, 0.827) | <0.001 |
| Cluster 3 vs Cluster 1 | WorkTime_3 | -0.759 | 0.174 | 18.937 | 0.468 (0.332, 0.659) | <0.001 |
| Cluster 3 vs Cluster 1 | WorkTime_4 | -0.395 | 0.289 | 1.865 | 0.674 (0.382, 1.188) | 0.172 |
| Cluster 3 vs Cluster 1 | SleepQuality_2 | -0.909 | 0.116 | 61.228 | 0.403 (0.321, 0.506) | <0.001 |
| Cluster 3 vs Cluster 1 | SleepQuality_3 | -1.872 | 0.178 | 110.038 | 0.154 (0.108, 0.218) | <0.001 |
| Cluster 3 vs Cluster 1 | Alcohol_2 | 0.309 | 0.350 | 0.779 | 1.362 (0.686, 2.706) | 0.378 |
| Cluster 3 vs Cluster 1 | Smoke_2 | -0.054 | 0.400 | 0.018 | 0.947 (0.433, 2.073) | 0.892 |
| Cluster 3 vs Cluster 1 | Exercise_2 | -1.313 | 0.096 | 187.625 | 0.269 (0.223, 0.325) | <0.001 |
| Cluster 3 vs Cluster 1 | ChronicDisease_2 | 0.774 | 0.323 | 5.750 | 2.168 (1.152, 4.081) | 0.016 |

**Note.** The multinomial model used Class 1 as the reference group and was interpreted as a sensitivity analysis for class-specific contrasts. The pattern generally supported the ordered-logistic findings, with stronger contrasts for Class 3 versus Class 1 than for Class 2 versus Class 1.

**Supplementary Table S6. Hospital-cluster robust ordered logistic sensitivity analysis**

| **Variable** | **Standard OR (95% CI)** | **Standard P** | **Hospital-cluster robust OR (95% CI)** | **Hospital-cluster robust P** | **Significance changed** |
| --- | --- | --- | --- | --- | --- |
| Age | 0.999 (0.940, 1.060) | 0.963 | 0.999 (0.932, 1.070) | 0.968 | No |
| FamilyMember | 0.976 (0.926, 1.030) | 0.380 | 0.976 (0.918, 1.039) | 0.453 | No |
| FamilyIncome | 0.968 (0.917, 1.022) | 0.236 | 0.968 (0.898, 1.043) | 0.395 | No |
| Income | 0.900 (0.849, 0.954) | <0.001 | 0.900 (0.845, 0.960) | 0.001 | No |
| DayShift | 1.044 (0.989, 1.102) | 0.115 | 1.044 (0.986, 1.106) | 0.140 | No |
| NightShift | 1.092 (1.034, 1.152) | 0.001 | 1.092 (1.023, 1.165) | 0.008 | No |
| SleepTime | 0.942 (0.891, 0.997) | 0.040 | 0.942 (0.884, 1.005) | 0.069 | Yes |
| Gender_2 | 1.100 (0.884, 1.370) | 0.392 | 1.100 (0.879, 1.378) | 0.404 | No |
| Rural_2 | 0.934 (0.826, 1.057) | 0.281 | 0.934 (0.830, 1.052) | 0.262 | No |
| LeftBy_2 | 0.845 (0.759, 0.939) | 0.002 | 0.845 (0.745, 0.958) | 0.008 | No |
| Religion_2 | 1.014 (0.811, 1.267) | 0.906 | 1.014 (0.799, 1.286) | 0.912 | No |
| Married_2 | 0.780 (0.634, 0.959) | 0.018 | 0.780 (0.633, 0.960) | 0.019 | No |
| Married_3 | 1.154 (0.494, 2.699) | 0.741 | 1.154 (0.506, 2.630) | 0.733 | No |
| Level_2 | 0.840 (0.721, 0.978) | 0.025 | 0.840 (0.678, 1.040) | 0.109 | Yes |
| Education_2 | 0.750 (0.538, 1.045) | 0.089 | 0.750 (0.520, 1.080) | 0.122 | No |
| Education_3 | 1.062 (0.749, 1.506) | 0.736 | 1.062 (0.718, 1.570) | 0.763 | No |
| Exposure_2 | 0.604 (0.543, 0.672) | <0.001 | 0.604 (0.558, 0.655) | <0.001 | No |
| WorkTime_2 | 1.259 (1.115, 1.422) | <0.001 | 1.259 (1.073, 1.478) | 0.005 | No |
| WorkTime_3 | 1.570 (1.300, 1.895) | <0.001 | 1.570 (1.243, 1.982) | <0.001 | No |
| WorkTime_4 | 1.352 (0.973, 1.880) | 0.072 | 1.352 (0.915, 2.000) | 0.130 | No |
| SleepQuality_2 | 1.751 (1.524, 2.012) | <0.001 | 1.751 (1.507, 2.034) | <0.001 | No |
| SleepQuality_3 | 3.521 (2.912, 4.257) | <0.001 | 3.521 (2.888, 4.292) | <0.001 | No |
| Alcohol_2 | 0.854 (0.593, 1.231) | 0.398 | 0.854 (0.598, 1.220) | 0.386 | No |
| Smoke_2 | 1.152 (0.751, 1.769) | 0.516 | 1.152 (0.613, 2.165) | 0.659 | No |
| Exercise_2 | 2.234 (1.995, 2.502) | <0.001 | 2.234 (1.962, 2.543) | <0.001 | No |
| ChronicDisease_2 | 0.662 (0.494, 0.888) | 0.006 | 0.662 (0.504, 0.871) | 0.003 | No |

**Note.** The sensitivity analysis used 5682 nurses from 89 cleaned hospital clusters after excluding 157 low-confidence free-text hospital identifiers. Odds-ratio directions remained unchanged; two predictors changed nominal significance status after cluster-robust standard errors were applied: SleepTime and Level_2.

# S4. Residualized network sensitivity results

Residualized networks were estimated after adjusting each PID, PSR, and ADP indicator for covariates associated with profile membership. These analyses evaluate whether the profile-specific network findings were robust to demographic and work-related covariates.

**Supplementary Table S7. Global characteristics of residualized class-specific networks**

| **Class** | **Nodes** | **Nonzero edges** | **Density** | **Global strength** | **Mean absolute nonzero edge** | **Positive edge proportion** | **Negative edge proportion** |
| --- | --- | --- | --- | --- | --- | --- | --- |
| Class 1 | 36 | 228 | 0.362 | 16.221 | 0.071 | 0.340 | 0.022 |
| Class 2 | 36 | 231 | 0.367 | 15.500 | 0.067 | 0.357 | 0.010 |
| Class 3 | 36 | 204 | 0.324 | 16.554 | 0.081 | 0.313 | 0.011 |

**Note.** Network density equals the number of nonzero edges divided by 630 possible undirected edges among 36 nodes. Global strength is the sum of absolute edge weights. After covariate adjustment, Class 2 retained the weakest global strength, whereas Class 3 retained the highest mean absolute nonzero edge weight.

**Supplementary Table S8. Network Comparison Test results for residualized sensitivity networks**

| **Comparison** | **Global strength difference** | **Global strength p** | **Maximum edge difference** | **Network invariance p** |
| --- | --- | --- | --- | --- |
| Class 1 vs Class 2 | 0.720 | 0.019 | 0.110 | 0.138 |
| Class 1 vs Class 3 | 0.334 | 0.447 | 0.175 | 0.031 |
| Class 2 vs Class 3 | 1.054 | <0.001 | 0.136 | 0.262 |

**Note.** Residualized NCT results were consistent with the main pattern: Class 1 versus Class 2 and Class 2 versus Class 3 differed in global strength, while Class 1 versus Class 3 showed evidence of network-structure difference without a significant global-strength difference.

**Supplementary Table S9. Top residualized bridge expected influence nodes by class**

| **Class** | **Rank** | **Node** | **Brief item meaning** | **Bridge strength** | **Bridge expected influence** |
| --- | --- | --- | --- | --- | --- |
| Class 1 | 1 | PSR2 | Achievement pride | 0.313 | 0.249 |
| Class 1 | 2 | PID8 | Social interaction | 0.247 | 0.247 |
| Class 1 | 3 | PID2 | Social confidence | 0.230 | 0.180 |
| Class 1 | 4 | PSR1 | Problem solving | 0.157 | 0.157 |
| Class 1 | 5 | PSR3 | Calm handling | 0.147 | 0.147 |
| Class 2 | 1 | PID2 | Social confidence | 0.370 | 0.302 |
| Class 2 | 2 | PSR3 | Calm handling | 0.247 | 0.247 |
| Class 2 | 3 | PID8 | Social interaction | 0.206 | 0.206 |
| Class 2 | 4 | PSR2 | Achievement pride | 0.190 | 0.190 |
| Class 2 | 5 | ADP4 | Clear thinking | 0.181 | 0.167 |
| Class 3 | 1 | PID2 | Social confidence | 0.301 | 0.293 |
| Class 3 | 2 | PSR5 | Control ability | 0.242 | 0.242 |
| Class 3 | 3 | PID8 | Social interaction | 0.217 | 0.217 |
| Class 3 | 4 | PSR2 | Achievement pride | 0.201 | 0.201 |
| Class 3 | 5 | PSR3 | Calm handling | 0.203 | 0.175 |

**Note.** Bridge expected influence was calculated across the PID, PSR, and ADP communities. Larger values indicate stronger cross-domain connecting roles after covariate adjustment. The strongest bridge nodes were concentrated mainly in professional identity and psychological resilience indicators.

**Supplementary Figure S1. Residualized Class 1 network**

**Note.** The network shows conditional associations among PID, PSR, and ADP indicators after covariate adjustment in the resource-constrained adaptive profile.

**Supplementary Figure S2. Residualized Class 2 network**

**Note.** The moderate resource-adaptation profile retained the weakest global strength after adjustment.

**Supplementary Figure S3. Residualized Class 3 network**

**Note.** The high resource-adaptation profile retained fewer but stronger nonzero edges after adjustment.

**Supplementary Figure S4. Top bridge expected influence nodes in residualized networks**

**Note.** The strongest cross-domain bridge nodes remained concentrated mainly in professional identity and psychological resilience indicators.

**Supplementary Figure S5. Complete standard centrality comparison for residualized networks**

**Note.** The figure provides a complete descriptive comparison of node-level strength and expected influence after covariate adjustment.

**Supplementary Figure S6. Complete bridge centrality comparison for residualized networks**

**Note.** The figure provides the full bridge-centrality profile for all nodes after covariate adjustment.

# S5. Centrality stability of base networks

Case-dropping bootstrap analyses were conducted to evaluate the stability of standard centrality and bridge centrality estimates in the base networks.

**Supplementary Table S10. CS coefficients for standard and bridge centrality in base networks**

| **Analysis** | **Class** | **N** | **CS strength** | **CS expected influence** | **CS bridge strength** | **CS bridge expected influence** | **Interpretation** |
| --- | --- | --- | --- | --- | --- | --- | --- |
| Base network | Class 1 | 1906 | 0.750 | 0.750 | 0.672 | 0.594 | Stable for standard and bridge centrality |
| Base network | Class 2 | 3152 | 0.750 | 0.750 | 0.750 | 0.750 | Stable for standard and bridge centrality |
| Base network | Class 3 | 781 | 0.750 | 0.750 | 0.594 | 0.594 | Stable for standard and bridge centrality |

**Note.** All standard and bridge CS coefficients exceeded 0.50, supporting stable interpretation of strength, expected influence, bridge strength, and bridge expected influence in the base networks.

**Supplementary Figure S7. Case-dropping bootstrap stability for standard centrality in the base Class 1 network**

**Note.** Lines show average correlations between centrality estimates from case-dropped samples and the original network estimates.

**Supplementary Figure S8. Case-dropping bootstrap stability for standard centrality in the base Class 2 network**

**Note.** Lines show average correlations between centrality estimates from case-dropped samples and the original network estimates.

**Supplementary Figure S9. Case-dropping bootstrap stability for standard centrality in the base Class 3 network**

**Note.** Lines show average correlations between centrality estimates from case-dropped samples and the original network estimates.

**Supplementary Figure S10. Case-dropping bootstrap stability for bridge centrality in the base Class 1 network**

**Note.** Lines show average correlations between bridge-centrality estimates from case-dropped samples and the original network estimates.

**Supplementary Figure S11. Case-dropping bootstrap stability for bridge centrality in the base Class 2 network**

**Note.** Lines show average correlations between bridge-centrality estimates from case-dropped samples and the original network estimates.

**Supplementary Figure S12. Case-dropping bootstrap stability for bridge centrality in the base Class 3 network**

**Note.** Lines show average correlations between bridge-centrality estimates from case-dropped samples and the original network estimates.

**Supplementary Figure S13. Edge-weight bootstrap confidence intervals for the base Class 1 network**

**Supplementary Figure S14. Edge-weight bootstrap confidence intervals for the base Class 2 network**

**Supplementary Figure S15. Edge-weight bootstrap confidence intervals for the base Class 3 network**

Overall, the base-network bootstrap results support cautious interpretation of individual edges while indicating that the broader centrality pattern was stable enough for profile-level comparison.

# S6. Centrality stability of residualized networks

Case-dropping bootstrap analyses were repeated for the residualized networks to evaluate whether standard and bridge centrality estimates remained stable after covariate adjustment.

**Supplementary Table S11. CS coefficients for standard and bridge centrality in residualized networks**

| **Analysis** | **Class** | **N** | **CS strength** | **CS expected influence** | **CS bridge strength** | **CS bridge expected influence** | **Interpretation** |
| --- | --- | --- | --- | --- | --- | --- | --- |
| Residualized network | Class 1 | 1906 | 0.750 | 0.750 | 0.672 | 0.594 | Stable for standard and bridge centrality |
| Residualized network | Class 2 | 3152 | 0.750 | 0.750 | 0.750 | 0.750 | Stable for standard and bridge centrality |
| Residualized network | Class 3 | 781 | 0.750 | 0.750 | 0.594 | 0.594 | Stable for standard and bridge centrality |

**Note.** The residualized networks showed the same stability profile as the base networks. All standard and bridge CS coefficients exceeded 0.50, supporting stable interpretation after covariate adjustment.

**Supplementary Figure S16. Case-dropping bootstrap stability for standard centrality in the residualized Class 1 network**

**Note.** Lines show average correlations between centrality estimates from case-dropped samples and the original residualized network estimates.

**Supplementary Figure S17. Case-dropping bootstrap stability for standard centrality in the residualized Class 2 network**

**Supplementary Figure S18. Case-dropping bootstrap stability for standard centrality in the residualized Class 3 network**

**Supplementary Figure S19. Case-dropping bootstrap stability for bridge centrality in the residualized Class 1 network**

**Supplementary Figure S20. Case-dropping bootstrap stability for bridge centrality in the residualized Class 2 network**

**Supplementary Figure S21. Case-dropping bootstrap stability for bridge centrality in the residualized Class 3 network**

**Supplementary Figure S22. Edge-weight bootstrap confidence intervals for the residualized Class 1 network**

**Supplementary Figure S23. Edge-weight bootstrap confidence intervals for the residualized Class 2 network**

**Supplementary Figure S24. Edge-weight bootstrap confidence intervals for the residualized Class 3 network**
